# Complete genome characterization, phylogenetic analysis, and capsid P2 variation of a goose astrovirus genotype 2 isolate from Guizhou, China

**DOI:** 10.1016/j.psj.2026.107662

**Published:** 2026-08-19

**Authors:** Qiufan Xu, Lijin Lai, Ting Li, Yong Pan, Li Yang, Jinge Xu

**Affiliations:** a Institute of Animal Husbandry and Veterinary Medicine, Guizhou Academy of Agricultural Sciences, Guiyang, Guizhou, China; b College of Veterinary Medicine, South China Agricultural University, Guangzhou, Guangdong, China

**Keywords:** Complete genome, Phylogenetic analysis, Capsid P2, Goose astrovirus genotype 2

## Abstract

Goose astrovirus genotype 2 (GAstV-2) is associated with gout and renal disease in goslings, but its occurrence in Guizhou Province remains poorly documented. We isolated a GAstV-2 strain, designated GZJP2024, from goslings with visceral gout on a farm in Jinping County, Guizhou Province, China. PCR detected GAstV-2 but not goose parvovirus, goose reovirus, Tembusu virus, fowl adenovirus, or goose astrovirus genotype 1. Serial passage in goose embryos produced mortality and hemorrhagic lesions during the third passage. Whole-genome sequencing yielded a 7,251-nt genome containing three overlapping open reading frames (ORF1a, ORF1b, and ORF2). Sequence identity and phylogenetic analyses assigned GZJP2024 to the GAstV-2 lineage. ORF1b was the most conserved coding region, whereas ORF2 was more variable. Comparison with consensus sequences from representative GAstV-2 strains identified five amino acid substitutions in ORF1a and ten in ORF2. Four ORF2 substitutions (E456D, L540Q, S608T, and A614T) occurred in the capsid P2 domain and overlapped or neighbored predicted B-cell epitope-rich regions. Template-based mapping placed E456D, S608T, and A614T on exposed regions of a spike-like capsid structure. These findings document a GAstV-2 isolate from a gout-affected goose farm in Guizhou and provide sequence data for future regional surveillance.

## Introduction

Avian astroviruses (AAstVs) are non-enveloped, positive-sense, single-stranded RNA viruses in the family Astroviridae. Their genomes are approximately 6.1-7.9 kb long and contain three open reading frames: ORF1a, ORF1b, and ORF2. These regions encode nonstructural proteins, an RNA-dependent RNA polymerase, and the capsid protein. Avian astroviruses cause enteric disease and are associated with nephritis and impaired growth in young poultry. Since 2016, goose astrovirus (GAstV) has emerged as an important pathogen of goslings in China, where it is associated with visceral gout, urate deposition, and high mortality. Although goose production is expanding in Guizhou Province, etiological surveillance and genomic data for local GAstV cases remain limited (Liu et al., 2022).

Etiological and phylogenetic studies have characterized GAstV-associated gout since the first virus isolation reports in 2018 (Zhang et al., 2018a; Jin et al., 2018; Yang et al., 2018). Capsid-based phylogeny divides GAstV into genotypes 1 and 2, with GAstV-2 predominating among gout-associated cases in China. Genomic surveys have identified extensive viral diversity, characteristic amino acid substitutions, and recombination signals in ORF1a and ORF2 (Cui et al., 2022; Fei et al., 2022). A study of 112 isolates collected during 2014-2024 further confirmed the predominance of GAstV-2 but showed sparse sampling in southwestern China (Li et al., 2025b). GAstV-2 has also been detected in ducks, indicating that its host range extends beyond geese (Chen et al., 2020; Wei et al., 2020b).

No GAstV isolate or complete genome from Guizhou Province has been publicly reported. This geographic gap limits understanding of GAstV-2 distribution and evolution in China. In addition, variation among ORF1a, ORF1b, and ORF2 and the potential antigenic relevance of capsid substitutions remain incompletely characterized. Analysis of isolates from Guizhou would therefore extend current genomic surveillance and provide an initial basis for broader regional studies.

In this study, we isolated a GAstV-2 strain from goslings with visceral gout on a farm in Guizhou Province and determined its complete genome sequence. We assessed embryo propagation, sequence identity, and phylogenetic relationships based on the complete genome and individual ORFs. We also characterized amino acid substitutions in ORF1a and ORF2 and evaluated predicted antigenic features of the capsid P2 domain.

## Materials and methods

### Ethics statement

Clinical samples were collected from goslings that died naturally on a commercial goose farm. No live animals were experimentally infected or euthanized for this study. Goose embryos were used only for virus isolation and propagation. Formal approval for animal experimentation was therefore not required under institutional guidelines. All sample collection and laboratory procedures complied with applicable biosafety and animal welfare requirements.

### Sample collection and viral nucleic acid detection

In 2024, nine goslings that died with clinical signs of gout were collected from a commercial farm in Guizhou Province, China. Liver and kidney tissues from each bird were pooled and homogenized. Total RNA and DNA were extracted separately. The homogenates were tested for fowl adenovirus (FAdV), goose parvovirus (GPV), Tembusu virus (TMUV), goose reovirus (GRV), GAstV-1, and GAstV-2 using the primers listed in Table 1.

**Table 1:** Primers used for viral gene detection in clinical samples.

Primer name	Sequence(5′→3′)	Product size (bp)	Reference
GAstV-1-F	TTCGCTGAGACTCCTATGTCA	119	(Zhang et al., 2022a)
GAstV-1-R	GCCCAACTTCTGGTAGCTTGAT		
GAstV-2-F	GCCTCTCTTCTGGCGGATAC	284	This study
GAstV-2-R	CCCTGAGTAACCTGAGCGTC		
GPV-F	CCAAGCTACAACCACAT	539	(Corrand et al., 2011)
GPV-R	TGAGCGAACATGCTATGGAAGG		
GRV-F	TGAGACGCCTGACTACGATT	380	(Zhang et al., 2018b)
GRV-R	ATGCTTGGAGTGAGACGACT		
FAdV-F	CAACTACATCGGGTTCAGGGATAACTTC	766	(Islam et al., 2026)
FAdV-R	CCAGTTTCTGTGGTGGTTGAAGGGGTT		
TMUV-F	GCCACGGAATTAGCGGTTGT	401	(Zhang et al., 2018b)
TMUV-R	TAATCCTCCATCTCAGCGGTGTAG		

### Virus isolation and identification

Virus isolation was attempted using samples positive only for GAstV-2. Kidney and liver tissues were homogenized in sterile phosphate-buffered saline (PBS; pH 7.4) to prepare a 20% (w/v) suspension, which was centrifuged at 8,000 × g for 10 min at 4 °C. For each passage, ten 10-day-old goose embryos were inoculated with 0.2 mL of the prepared inoculum through the chorioallantoic cavity and incubated at 37.8 °C and 55% relative humidity. Deaths within 24 h were considered nonspecific and excluded. Embryos that died after 24 h or survived to 5 days post-inoculation were chilled overnight at 4 °C. Allantoic fluid was harvested and used as inoculum for the next passage. After three passages, allantoic-fluid nucleic acids were tested for GAstV-2, GAstV-1, FAdV, GPV, TMUV, and GRV using the primers in Table 1.

### Complete genome amplification and sequencing

The initial amplicon was sequenced and analyzed using BLAST to identify the most similar reference genome. Conserved terminal sequences from that genome were used to design primers for complete amplification of ORF1a, ORF1b, and ORF2 (Table 2). PCR products were cloned into the pCE3 Blunt Vector (Vazyme, Nanjing, China) and sequenced by the Sanger method. Third-passage GAstV-2-positive allantoic fluid was also submitted to Sangon Biotech (Shanghai, China) for whole-genome sequencing.

**Table 2:** Primers used for GAstV-2 ORF amplification.

Primer name	Sequence(5′→3′)	Product size (bp)
ORF1a-F	ATGGCGGCCGGTGGCCC	3255
ORF1a-R	CTAGTTTTTTCCACACGTTTCACG	
ORF1b-F	AAAAAACTAGATAAGGGGGACGATG	1551
ORF1b-R	TCACCAGATTTGAGAAAAGAAGTCG	
ORF2-F	ATGGCAGACAGGGCGGTG	2115
ORF2-R	TCACTCATGTCCGCCCTTCTC	

### Whole-genome structure analysis

Transmembrane domains (TM) in ORF1a were predicted using TMHMM (https://services.healthtech.dtu.dk/services/TMHMM-2.0/). The protease region (Pro) was identified using NCBI (https://www.ncbi.nlm.nih.gov/Structure/cdd/wrpsb.cgi). Nuclear localization sequence (NLS) was predicted with cNLS Mapper (http://nls-mapper.iab.keio.ac.jp/cgi-bin/NLS_Mapper_form.cgi).

### Sequence alignments and phylogenetic analysis

Reference nucleotide and amino acid sequences for GAstV and other avian astroviruses were retrieved from GenBank. Selected avian nephritis virus and mammalian astrovirus sequences served as outgroups. The GZJP2024 complete genome was aligned with the reference genomes, and a maximum-likelihood tree was inferred using the GTR+F+R5 model with 10,000 ultrafast bootstrap replicates. Separate maximum-likelihood analyses of ORF1a, ORF1b, and ORF2 amino acid sequences used 10,000 ultrafast bootstrap replicates. The LG+I+G4 model was applied to ORF1a and ORF1b, and Q.YEAST+F+I+G4 was applied to ORF2.

Sequence alignment, trimming, and phylogenetic analyses were performed in PhyloSuite v2 (Zhao et al., 2025). Trees were visualized using Interactive Tree of Life (iTOL) v6 (Letunic and Bork, 2024).

### Amino acid variation and consensus sequence analysis

The ORF1a and ORF2 amino acid sequences of 20 GAstV-2 reference strains used in the phylogenetic analyses were aligned. At each position, the most frequent residue was designated the GAstV-2 consensus amino acid. A characteristic substitution was recorded when the GZJP2024 residue differed from this modal residue. Substitutions are written as consensus residue-position-GZJP2024 residue. Accession numbers and metadata for the reference strains are provided in Supplementary Table S1.

### Epitope prediction and structural mapping of the capsid P2 domain

The GZJP2024 P2 domain was defined as ORF2 residues 425-665 according to the NCBI Conserved Domain Database. Immune-related properties were evaluated using the Parker hydrophilicity, Karplus-Schulz flexibility, Emini surface accessibility, and Kolaskar-Tongaonkar antigenicity scales. Residues positive in at least three analyses were considered candidate immune-relevant residues, and continuous positive stretches of at least six residues were retained as candidate antigenic segments. B-cell epitopes were predicted using BepiPred-3.0 at its default threshold of 0.1512. Threshold-based calls, top-20% epitope candidates, and top-20% linear-epitope candidates were mapped to P2 coordinates. Canonical N-linked glycosylation sequons were identified as N-X-S/T motifs, where X is any residue except proline. For structural context, the P2 sequence was aligned to the experimentally resolved porcine astrovirus spike structure 7XCA from the RCSB Protein Data Bank. BepiPred-supported regions and substitutions E456D, L540Q, S608T, and A614T were projected onto the aligned template.

## Results

### Identification and isolation of GAstV from samples

**Fig. 1:**
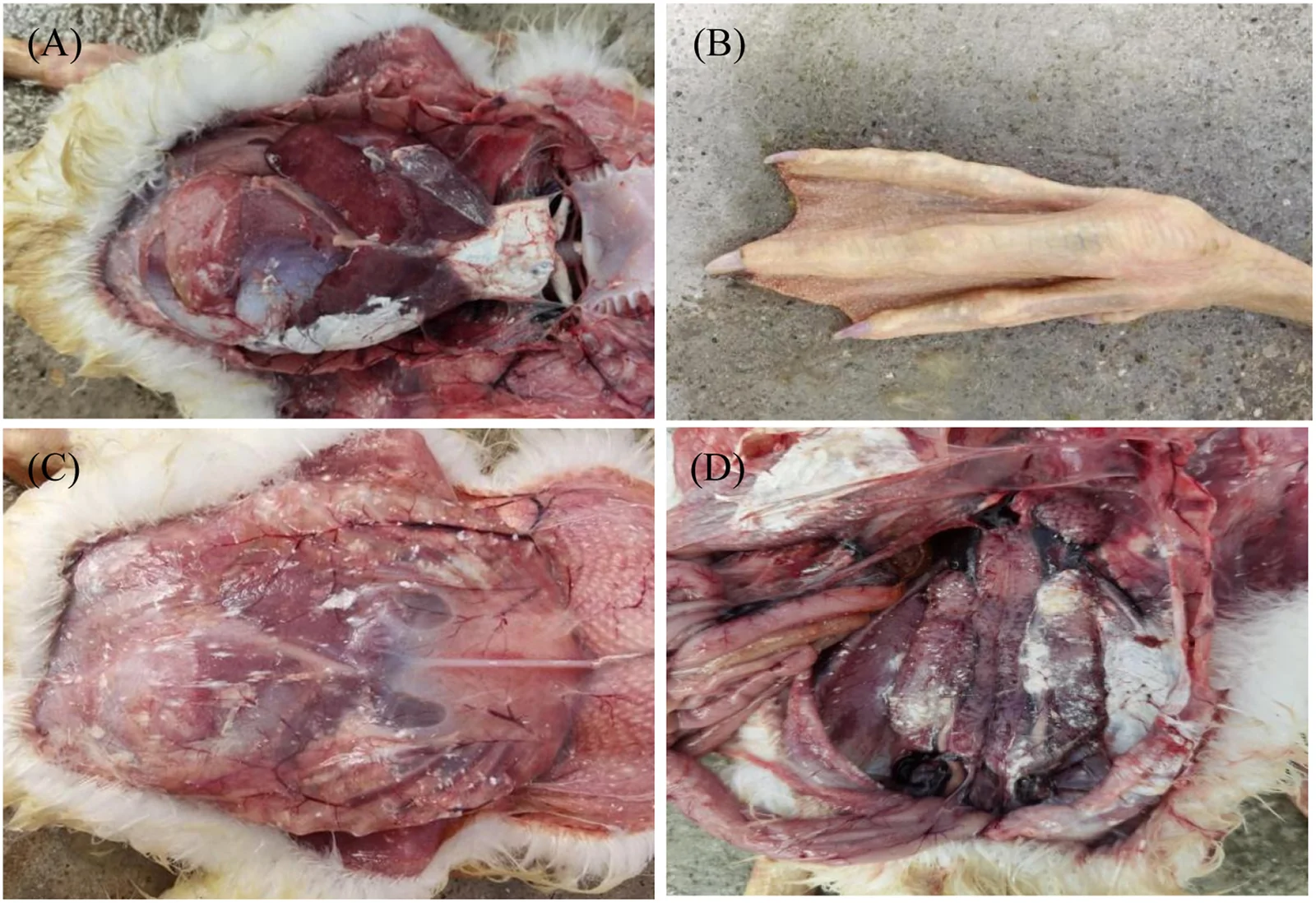
Gross lesions in the sampled goslings. (A) White urate deposits on the surface of the heart and liver. (B) Urate deposits on the toes. (C) White urate deposits on the skin. (D) Severe urate deposition in the abdominal cavity.

**Fig. 2:**
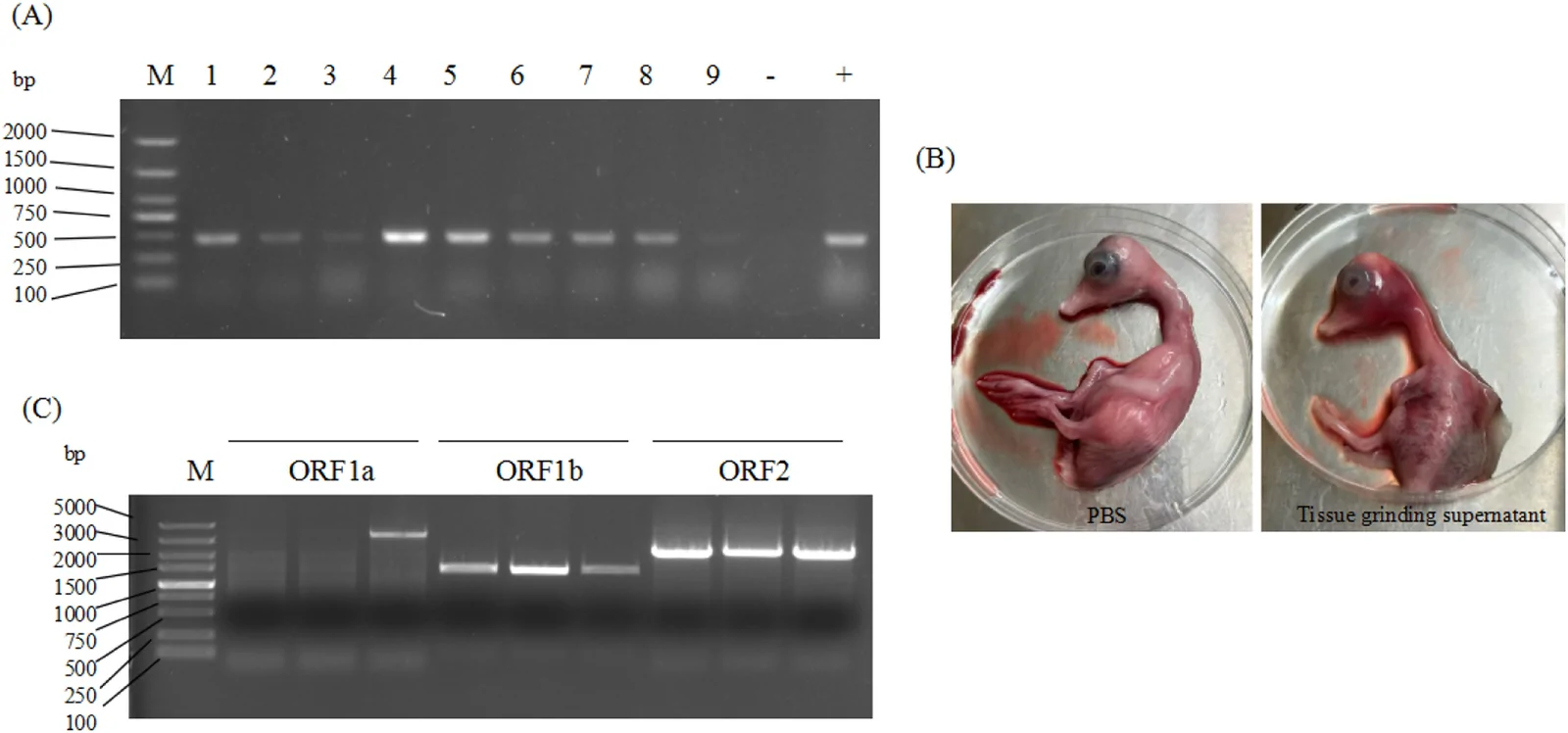
Detection and embryo propagation of GZJP2024. (A) RT-PCR detection of GAstV-2 in pooled liver and kidney homogenates from nine dead goslings. (B) Hemorrhage in a goose embryo inoculated with the third-passage isolate at 4 days post-inoculation. (C) Amplification of ORF1a, ORF1b, and ORF2 from cDNA prepared using third-passage allantoic-fluid RNA.

In 2024, gosling mortality occurred on a farm in Jinping County, Qiandongnan Prefecture, Guizhou Province. Affected birds showed clinical signs of gout, and necropsy revealed urate deposits on the liver, heart, and toes (Fig. 1). PCR detected only GAstV-2; GPV, GRV, TMUV, FAdV, and GAstV-1 were not detected (Fig. 2A). Positive samples were inoculated into 10-day-old goose embryos. No mortality occurred during passages 1 or 2. During passage 3, embryos died at 4 days post-inoculation and showed gross hemorrhagic lesions (Fig. 2B). The isolate was designated GZJP2024.

### Complete genome characterization

High-throughput sequencing and Sanger sequencing of the three ORFs produced identical sequences, supporting the assembled genome (Fig. 2C). After quality control, 69,166,954 clean reads were retained, with 96.93% of bases at Q30 or higher. De novo assembly generated one 7,251-nt viral contig with a mean depth of 244.69× and 100% genome coverage. BLAST analysis showed high similarity to GAstV-2 reference sequences, including MF772821.1. No other avian viral sequences were detected.

**Fig. 3:**
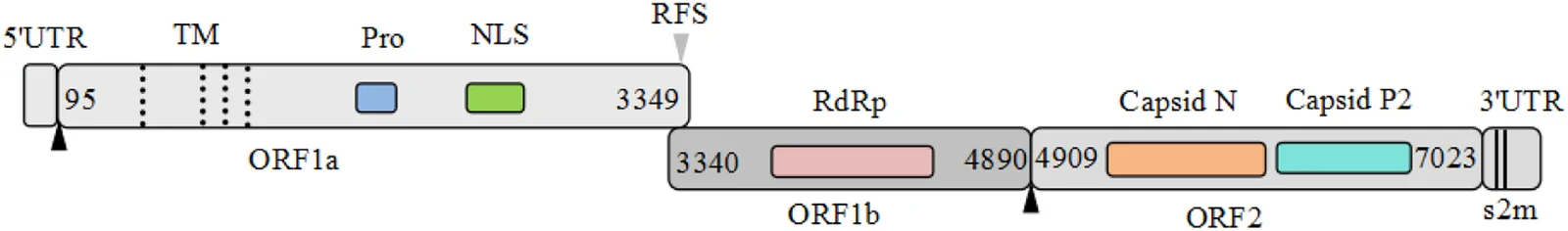
Predicted genome organization of GZJP2024. Nucleotide coordinates include stop codons. Black triangles indicate the translation start sites of ORF1a and ORF2; the gray triangle indicates the AAAAAAC ribosomal frameshift signal. Abbreviations: NLS, nuclear localization signal; ORF, open reading frame; Pro, protease; RdRp, RNA-dependent RNA polymerase; RFS, ribosomal frameshift signal; s2m, stem-loop II-like motif; TM, transmembrane domain; Capsid N, N-terminal domain of the capsid protein; Capsid P2, protruding domain 2 of the capsid protein.

The GZJP2024 genome comprised 7,251 nt and contained three overlapping ORFs: ORF1a (3,255 nt), ORF1b (1,551 nt), and ORF2 (2,115 nt). It also contained a 94-nt 5′ untranslated region, a 228-nt 3′ untranslated region, and a 22-nt poly(A) tail (Fig. 3).

ORF1a (nt 95-3349) encoded a 1,084-aa nonstructural polyprotein containing a trypsin-like peptidase domain (aa 561-688), a nuclear localization signal (aa 881-889; YQVKKRKTF), and four predicted transmembrane domains (aa 231-248, 384-406, 421-443, and 455-477). ORF1b (nt 3340-4890) encoded a 516-aa RNA-dependent RNA polymerase containing the conserved G_327_NPSGQYSTTVDNN, Y_377_GDD, and F_405_GMWVK motifs. The ORF1a-ORF1b overlap contained the heptameric ribosomal frameshift signal AAAAAAC at nt 3340-3346. ORF2 (nt 4909-7023) encoded the capsid precursor. A stem-loop II-like motif was identified at nt 7034-7076 in the 3′ untranslated region (Jonassen et al., 1998).

### Genetic and phylogenetic analyses

We compared the complete genome and individual ORFs of GZJP2024 with representative avian astroviruses, including GAstV-1, GAstV-2, turkey astrovirus 1 and 2, duck astrovirus 1-4, chicken astrovirus, and avian nephritis virus. Complete-genome nucleotide identity ranged from 50.75% with avian nephritis virus to 97.97% with GAstV-2 (Table 3). Amino acid identities ranged from 27.25% to 99.45% for ORF1a, 12.79% to 99.61% for ORF1b, and 27.06% to 99.15% for ORF2. GZJP2024 shared its highest identity with GAstV-2 in all comparisons. Among GAstV-2 comparisons, ORF1b was the most conserved region and ORF2 the most variable.

**Table 3:** Sequence identity between GZJP2024 and representative members of the genus AAstV.

AAstV Species	Accession No	Sequence identity (%)			
		Genome	ORF1a	ORF1b	ORF2
GAstV-1	OK571391	56.70%	47.50%	61.21%	40.59%
GAstV-2	OL963558	97.97%	99.45%	99.61%	99.15%
TAstV-1	NC_002470	53.67%	39.53%	12.79%	37.23%
TAstV-2	EU143843	61.64%	59.76%	68.74%	56.16%
DAstV-1	JX439643	59.58%	47.90%	64.27%	56.10%
DAstV-2	KF753806	60.78%	58.19%	67.96%	38.62%
DAstV-3	KJ020899	57.17%	49.63%	64.85%	36.43%
DAstV-4	JX624774	55.75%	41.81%	61.48%	34.69%
CAstV	KX397576	56.76%	47.93%	65.44%	36.00%
ANV	AB033998	50.75%	27.25%	51.47%	27.06%

ORF2 amino acid p-distances between GZJP2024 and representative avian astroviruses ranged from 0.008 to 0.594. The distance between GZJP2024 and the GAstV-2 reference strain OL963558 was 0.0085, whereas distances to turkey astrovirus 2 and duck astrovirus 1 were 0.438 and 0.439, respectively. These results supported the assignment of GZJP2024 to GAstV-2.

**Fig. 4:**
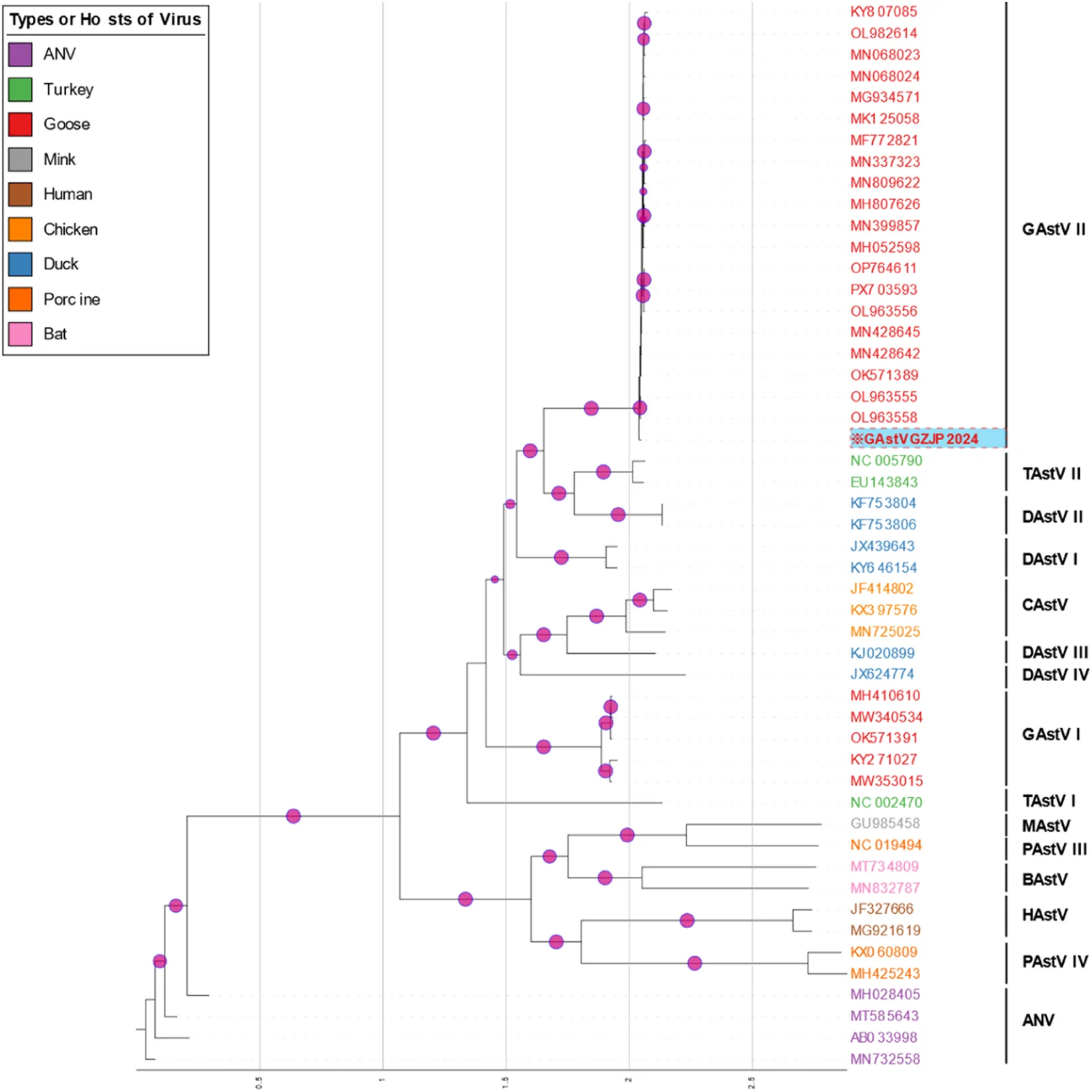
Maximum-likelihood phylogeny of GZJP2024 and representative astrovirus complete genomes retrieved from GenBank. The tree was inferred using the GTR+F+R5 model with 10,000 ultrafast bootstrap replicates.

**Fig. 5:**
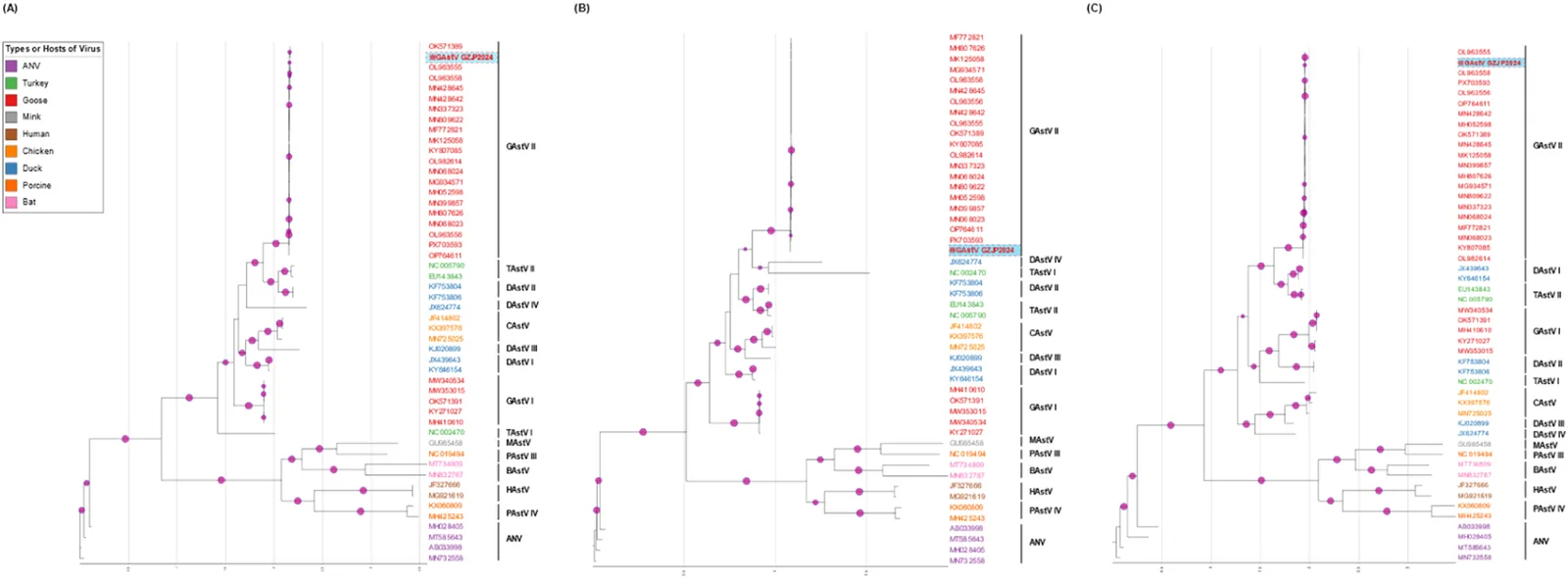
Maximum-likelihood phylogenies based on ORF1a (A), ORF1b (B), and ORF2 (C) amino acid sequences. The LG+I+G4 model was used for ORF1a and ORF1b, and Q.YEAST+F+I+G4 was used for ORF2. Each analysis used 10,000 ultrafast bootstrap replicates. GZJP2024 is marked in each tree.

Maximum-likelihood analysis placed GZJP2024 within the GAstV-2 lineage in the complete-genome tree (Fig. 4). ORF1a, ORF1b, and ORF2 amino acid trees yielded the same lineage assignment (Fig. 5A–C). No dedicated recombination analysis was performed. Together with the identity comparisons, these analyses identified ORF1b as the most conserved coding region and ORF2 as the most variable.

### Characteristic amino acid substitutions in ORF1a and ORF2

The ORF1a and ORF2 amino acid sequences of GZJP2024 were compared with modal consensus sequences derived from 20 representative GAstV-2 strains. ORF1b was excluded because it showed the highest amino acid conservation. Five ORF1a substitutions were identified: V555I, H589Y, A782T, S926T, and T975I (Table 4). These substitutions did not form an obvious cluster.

**Table 4:** Amino acid differences between GZJP2024 and modal consensus sequences derived from 20 GAstV-2 reference strains.

Protein	Position	GZJP2024	GAstV-2 consensus AA	Location
ORF1a	555	I	V	N/A
ORF1a	589	Y	H	Pro
ORF1a	782	T	A	N/A
ORF1a	926	T	S	N/A
ORF1a	975	I	T	N/A
ORF2	26	Q	H	N-terminal region
ORF2	228	T	A	Capsid N
ORF2	229	P	Q	Capsid N
ORF2	289	T	A	Capsid N
ORF2	380	S	N	Capsid N
ORF2	424	L	M	N/A
ORF2	456	D	E	Capsid P2
ORF2	540	Q	L	Capsid P2
ORF2	608	T	S	Capsid P2
ORF2	614	T	A	Capsid P2

Note: The GAstV-2 consensus amino acid is the most frequent residue at each aligned position among the 20 reference strains. A characteristic substitution was defined as a GZJP2024 residue that differed from this modal residue. Substitutions are written as consensus residue-position-GZJP2024 residue. Reference-strain metadata are provided in Supplementary Table S1.

Ten substitutions were identified in ORF2: H26Q, A228T, Q229P, A289T, N380S, M424L, E456D, L540Q, S608T, and A614T. The NCBI Conserved Domain Database assigned residues 47-404 to the capsid N domain and residues 425-665 to the P2 domain. Four substitutions occurred in the capsid N domain (A228T, Q229P, A289T, and N380S), and four occurred in P2 (E456D, L540Q, S608T, and A614T).

### Predicted antigenic features and structural localization of mutations in the capsid P2 domain

**Fig. 6:**
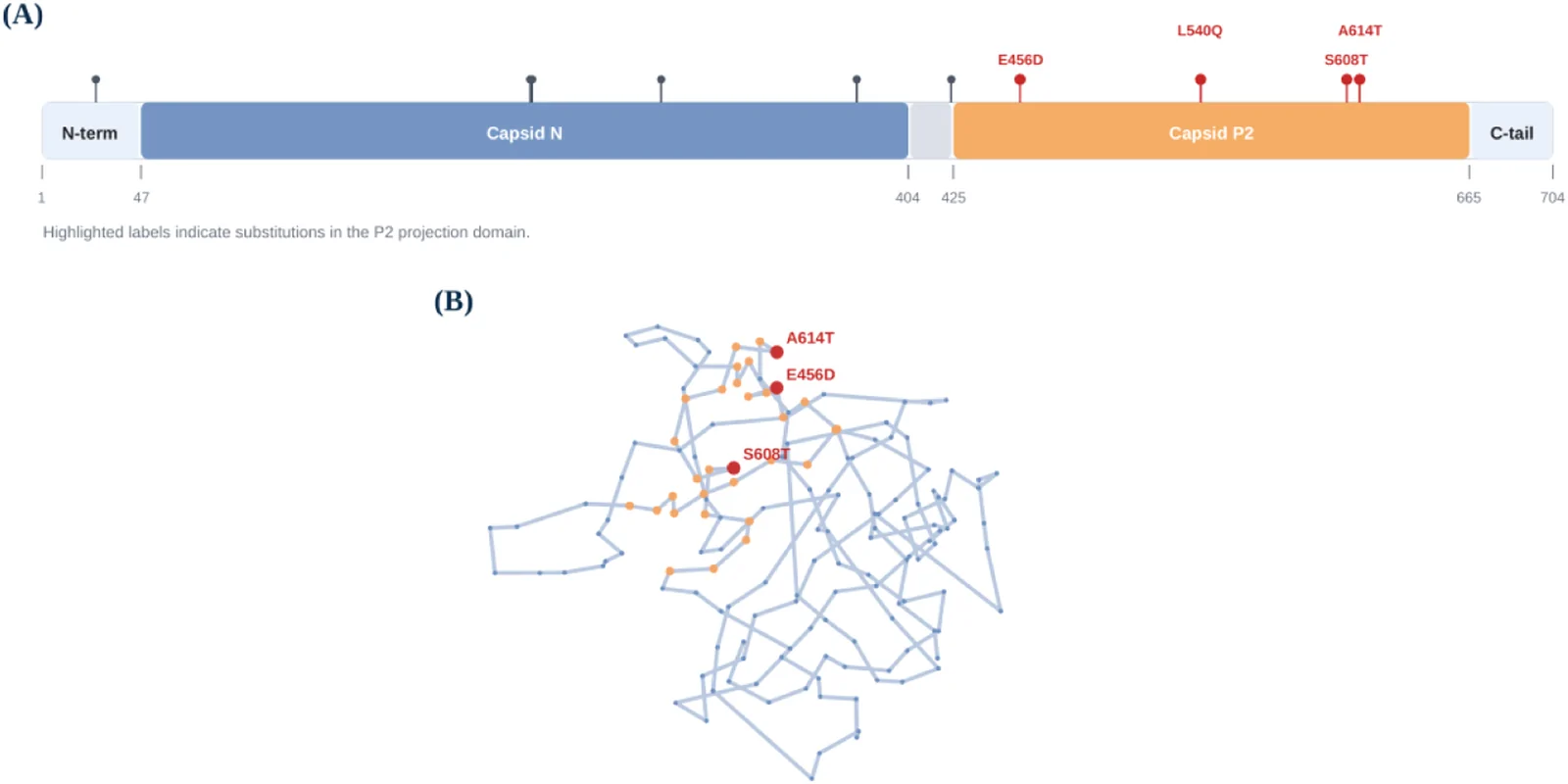
Predicted antigenic features and structural mapping of the GZJP2024 capsid P2 domain. (A) ORF2 domain organization and characteristic substitutions. Substitutions are written as GAstV-2 consensus residue-position-GZJP2024 residue. (B) Template-based mapping of GZJP2024 P2 onto the porcine astrovirus spike structure 7XCA. Blue indicates mapped P2 residues, orange indicates BepiPred-supported epitope-associated residues, and red indicates substitution sites. L540Q lacked a reliable template coordinate because it occurred within an alignment gap.

Sequence-property screening identified eight candidate antigenic segments at ORF2 residues 435-443, 454-462, 471-477, 531-537, 560-565, 585-591, 606-614, and 626-641. The 454-462 segment contained E456D, whereas the 606-614 segment contained S608T and A614T. Four canonical N-linked glycosylation sequons occurred at residues 464-467, 531-534, 565-568, and 602-605. Fig. 6A shows the substitutions relative to the capsid N and P2 domains.

BepiPred-3.0 predicted three epitope-rich regions containing characteristic substitutions: ORF2 residues 450-462, 531-543, and 602-615. E456D met the default threshold and was retained in both top-20% prediction sets. L540Q and S608T met the default threshold and were retained in the top-20% linear-epitope set. A614T met only the default threshold. Thus, all four P2 substitutions occurred within or adjacent to predicted epitope-rich regions.

The GZJP2024 P2 sequence shared 42.3% identity with the 7XCA astrovirus spike domain across 196 aligned positions. E456D, S608T, and A614T mapped to template residues 33, 208, and 214, respectively. L540Q occurred within an alignment gap and lacked a reliable template coordinate. The three mapped substitutions and BepiPred-supported regions occurred on exposed regions of the provisional structure (Fig. 6B).

## Discussion

GAstV-2 is frequently detected in goslings with gout and renal disease in China (Zhang et al., 2018b; Yang et al., 2018; Yuan et al., 2019). Here, we isolated GZJP2024 from goslings with visceral gout on a farm in Guizhou. The affected birds had multisystemic urate deposits, and PCR detected GAstV-2 but not five other tested goose viruses. High-throughput sequencing recovered a complete GAstV-2 genome and detected no other avian viral sequences. These findings document GAstV-2 in one investigated outbreak but do not establish its prevalence, distribution, or diversity across Guizhou Province.

GZJP2024 had the typical avian astrovirus genome organization, including three overlapping ORFs and conserved replication-associated features. Complete-genome and ORF-specific phylogenies assigned the isolate to GAstV-2. However, concordant lineage assignment cannot exclude recombination. Recombination has been reported in goose-origin astroviruses (Shen et al., 2022), and dedicated analyses with broader sequence sampling are needed to evaluate the recombination history of GZJP2024.

The sequence comparisons indicated different evolutionary constraints across the GAstV genome. ORF1b, which encodes the RNA-dependent RNA polymerase, was the most conserved region, whereas capsid-encoding ORF2 was more variable. This pattern is consistent with stronger functional constraint on the polymerase and greater host-related or immune pressure on the capsid. Because GAstV sequences remain geographically uneven across China, GZJP2024 adds useful genomic information from an underrepresented southwestern province (Fu et al., 2022; He et al., 2022; Li et al., 2025b).

GZJP2024 contained ten characteristic substitutions in ORF2 and five in ORF1a. Four ORF2 substitutions occurred in the P2 domain, a surface-exposed capsid region implicated in antigenicity and host interactions (Ykema and Tao, 2021; Haley et al., 2024). Previous studies have identified diagnostically and immunologically relevant B-cell epitopes in the GAstV-2 capsid protein (Ren et al., 2022; Zhang et al., 2022b; Li et al., 2025a, 2025c; Yuan et al., 2026). The P2 substitutions in GZJP2024 may therefore affect an antigenically relevant region, although this interpretation remains predictive.

BepiPred-3.0 identified three epitope-rich P2 regions that contained or neighbored E456D, L540Q, S608T, and A614T. Template-based mapping placed E456D, S608T, and A614T on exposed regions of a spike-like structure. Their accessibility suggests possible relevance to antibody recognition, but sequence prediction and homology-based mapping cannot demonstrate antigenic effects. Neutralization assays, monoclonal-antibody mapping, or peptide-based serology are required to determine whether these substitutions alter immune recognition.

GAstV transmission may extend across production stages and waterfowl hosts, as previous studies have reported vertical transmission and infections in ducks (Chen et al., 2020, 2021; Wei et al., 2020a, 2020b). GZJP2024 was isolated from affected goslings and propagated in goose embryos. Clinical findings, pathogen screening, virus isolation, and genomic evidence support an association with the investigated gout case. However, causality was not established because no controlled infection experiment was performed. Experimental infection studies are required to determine the pathogenicity of GZJP2024.

This study has three main limitations. First, it examined one outbreak and one isolate, precluding conclusions about GAstV-2 prevalence or diversity across Guizhou. Second, the antigenic interpretation relied on sequence-based predictions rather than immunological experiments. Third, structural mapping used a homologous astrovirus template rather than an experimentally resolved GZJP2024 capsid structure. Nevertheless, the study provides a complete genome, phylogenetic placement, domain-level variation analysis, and preliminary antigenic characterization of a GAstV-2 isolate from a gout-affected farm. GZJP2024 provides initial molecular evidence for GAstV-2 in the investigated case and supports broader surveillance across Guizhou Province.

## Author contributions

Qiufan Xu drafted the manuscript and acquired funding. Lijin Lai and Yong Pan performed the bioinformatics, epitope prediction, and structural analyses. Ting Li and Li Yang collected the samples and conducted virus isolation and sequencing. Jinge Xu revised the manuscript. All authors reviewed and approved the final manuscript.

## Disclosures

The authors declare that they have no known competing financial interests or personal relationships that could have appeared to influence the work reported in this paper.

## Footnotes

Supplementary material associated with this article can be found, in the online version, at doi:10.1016/j.psj.2026.107662.
