# Supplementary material for: Complete genome characterization, phylogenetic analysis, and capsid P2 variation of a goose astrovirus genotype 2 isolate from Guizhou, China
Source: Poult Sci. 2026 Aug 19;105(11):107662. doi: 10.1016/j.psj.2026.107662 (PMC13590105; doi:10.1016/j.psj.2026.107662)
Supplement: Supplementary file 1 [file mmc1.docx]

| GenBank accession | Strain/isolate | Host | Geographical origin | Collection year |
| --- | --- | --- | --- | --- |
| MF772821 | AAstV/Goose/CHN/2017/SD01 | Goose | China | 2017 |
| MH807626 | AstV/Goose/CXZ/18 | Goose | China | 2018 |
| MK125058 | JSHA | Goose | China | 2016 |
| MG934571 | GD | Goose | China | 2017 |
| OL963558 | JSW10 | Goose | China | 2020 |
| MN428645 | AHAU5 | Goose | China | 2018 |
| OL963556 | JSSQ | Goose | China | 2020 |
| MN428642 | GD AHAU2 | Goose | China | 2018 |
| OL963555 | JSCZ | Goose | China | 2020 |
| OK571389 | ZJLD | Goose | China | 2020 |
| KY807085 | HN1G | Goose | China | 2014 |
| OL982614 | JSCZ15 | Goose | China | 2015 |
| MN337323 | XX | Goose | China | 2018 |
| MN068024 | GTF-07 | Goose | China | 2018 |
| MN809622 | AstV/SDTA | Duck | China | 2020 |
| MH052598 | AstV/SDPY/Goose/1116/17 | Goose | China | 2017 |
| MN399857 | SDXT | Duck | China | 2019 |
| MN068023 | GTF-04 | Goose | China | 2018 |
| OP764611 | AH-16 | Goose | China | 2021 |
| PX703593 | AAstV/Goose/Shandong/D08/2022 | Goose | China | 2022 |

Supplementary Table S1. Detailed information on the GAstV-2 reference strains used for amino acid.
